# Chimeric Vaccines Designed by Immunoinformatics-Activated Polyfunctional and Memory T cells that Trigger Protection against Experimental Visceral Leishmaniasis

**DOI:** 10.3390/vaccines8020252

**Published:** 2020-05-27

**Authors:** Rory Cristiane Fortes De Brito, Jeronimo Conceição Ruiz, Jamille Mirelle de Oliveira Cardoso, Thais Lopes Valentim Di Paschoale Ostolin, Levi Eduardo Soares Reis, Fernando Augusto Siqueira Mathias, Rodrigo Dian de Oliveira Aguiar-Soares, Bruno Mendes Roatt, Rodrigo Corrêa-Oliveira, Daniela de Melo Resende, Alexandre Barbosa Reis

**Affiliations:** 1 Laboratório de Imunopatologia, Núcleo de Pesquisas em Ciências Biológicas/NUPEB, Universidade Federal de Ouro Preto, Ouro Preto 35400-000, Brazil; rorybrito@gmail.com (R.C.F.D.B.); ja_mirelle@yahoo.com.br (J.M.d.O.C.); thais.ostolin@gmail.com (T.L.V.D.P.O.); levieduardo@yahoo.com.br (L.E.S.R.); fa_mathias@yahoo.com.br (F.A.S.M.); rodrigodian@gmail.com (R.D.d.O.A.-S.); roatt@ufop.edu.br (B.M.R.); 2 Grupo Informática de Biossistemas e Genômica, Instituto René Rachou, Fiocruz Minas, Belo Horizonte 30190-002, Brazil; jeronimo.ruiz@fiocruz.br (J.C.R.); dani.melo.resende@gmail.com (D.d.M.R.); 3 Programa de Pós-graduação em Biologia Computacional e Sistemas, Instituto Oswaldo Cruz, Fiocruz, Rio de Janeiro 21040-360, Brazil; 4 Instituto Nacional de Ciência e Tecnologia em Doenças Tropicais (INCT-DT), Salvador 40110-160, Brazil; 5 Laboratório de Imunologia Celular e Molecular, Instituto René Rachou, Fiocruz Minas, Belo Horizonte 30190-002, Brazil; rodrigo.correa@fiocruz.br

**Keywords:** reverse vaccinology, immunoinformatics, chimera vaccine, Leishmania infantum, polyfunctional T cells, memory T cells, rational design of vaccines

## Abstract

Many vaccine candidates against visceral leishmaniasis (VL) have been proposed; however, to date, none of them have been efficacious for the human or canine disease. On this basis, the design of leishmaniasis vaccines has been constantly changing, and the use of approaches to select specific epitopes seems to be crucial in this scenario. The ability to predict T cell-specific epitopes makes immunoinformatics an even more necessary approach, as in VL an efficient immune response against the parasite is triggered by T lymphocytes in response to *Leishmania* spp. immunogenic antigens. Moreover, the success of vaccines depends on the capacity to generate long-lasting memory and polyfunctional cells that are able to eliminate the parasite. In this sense, our study used a combination of different approaches to develop potential chimera candidate vaccines against VL. The first point was to identify the most immunogenic epitopes of *Leishmania infantum* proteins and construct chimeras composed of Major histocompatibility complex (MHC) class I and II epitopes. For this, we used immunoinformatics features. Following this, we validated these chimeras in a murine model in a thorough memory study and multifunctionality of T cells that contribute to a better elucidation of the immunological protective mechanisms of polyepitope vaccines (chimera A and B) using multicolor flow cytometry. Our results showed that in silico-designed chimeras can elicit polyfunctional T cells producing T helper (Th)1 cytokines, a strong immune response against *Leishmania* antigen, and the generation of central and effector memory T cells in the spleen cells of vaccinated animals that was able to reduce the parasite burden in this organ. These findings contribute two potential candidate vaccines against VL that can be used in further studies, and help in this complex field of vaccine development against this challenging parasite.

## 1. Introduction

During the last decades, the way in which the immune system works and the protective mechanisms related to vaccination for complex parasites has been extensively interpreted, and the central dogma of vaccination has been mounted [1]. Some studies support the idea that an antigen is capable of inducing long-lasting immunological memory and protective immunity against re-challenge with the same pathogen. Although this concept has been successful in generating potent vaccines for many diseases, leishmaniasis caused by the protozoan parasite *Leishmania* remains a threatening exception. Thus, the design of leishmaniasis vaccines has been constantly changing, and the use of polyepitope vaccines seems to gain prominent space in this scenario. In one of the pioneering studies of visceral leishmaniasis (VL) polyepitope vaccines, a DNA vaccine containing *Leishmania donovani* GP63 protein T cell epitopes was proposed. The authors evaluated the immunogenicity of the vaccine in immunized and challenged BALB/c mice showing increased production of Interferon gama (IFN-γ) and Interleukin (IL)-2 in splenocytes of vaccinated animals. In addition, this vaccine reduced parasite load in the spleen and liver of challenged mice [2]. In light of this, seeking to expand the antigenic repertoire of vaccines, the authors of [3] constructed a multiepitope DNA vaccine that encoded four protein-fused peptides, lipophosphoglycan (LPG)-3, *Leishmania major* stress inducible protein (LmSTI)-1, cysteine peptidase B (CPB), and cysteine peptidase C (CPC). They evaluated the cytotoxic activity of lymphocytes and IFN-γ production in transgenic mouse (holding human MHC alleles, Human leukocyte antigen (HLA)-DRB1 * 0101/HLA-A * 0201), and the results revealed increased cytotoxic pleaseactivity and IFN-γ production after immunization [3]. Along the same line of studies, the authors of [4] constructed DNA vaccines based on peptides selected from *L. donovani* antigens (CPA, CPB, Kinetoplastid membrane protein (KMP)11, Thiol specific-antioxidant protein (TSA), and Elongation factor 1 (P74) that showed a significant reduction of parasite burden in the spleen after immunization using a unique preparation of these antigens as DNA vaccine when the mice were challenge with *L. donovani* promastigotes [4].

The basic assumption that introduction of an antigen into a host will generate protective immunity against the pathogen appears to be invalid. Thus, there are possible reasons for the failures and the possible approaches that may bring success to generation of *Leishmania* vaccine. First, the traffic of T cells between lymph nodes and the microenvironment on the site of infection is essential for activation and maturation of the right cells [1,5]. Besides this, the major challenge faced by the immunologists is how to identify antigens capable of generating long-lasting immunological memory. Therefore, several approaches to the evaluation of immunological memory have been developed using multicolor flow cytometry, which aims to identify and evaluate effector and memory T lymphocyte subpopulations to validate different vaccine candidates [6]. The ability to predict T cell-specific epitopes makes immunoinformatics an even more necessary approach, as in VL an efficient immune response against specific epitopes of the parasite is triggered by T lymphocytes in response to some *Leishmania* spp. [7,8,9]. Thus, some research groups have been proposing vaccine candidates on the basis of specific class I and II MHC-binding epitopes mapped to known proteins [3,4,10].

Therefore, the development of polyepitope vaccines is a promising field that has been studied in recent years. In this sense, our study used a combination of different approaches to develop candidate vaccines against VL. The first point was to identify the best tools to map immunogenic epitopes and construct chimeras composed of MHC class I and II epitopes. For this, we used immunoinformatics features described by [9]. Afterwards, we validated these chimeras in BALB/c mice in a thorough memory study and multifunctionality of T cells that contributes to a better elucidation of the immunological protective mechanisms of chimeras (A and B) constituted of polyepitope vaccines.

## 2. Materials and Methods

### 2.1. Ethical Statement

The study was performed according to the recommendation of the National Institute of Health, USA. The protocol number 2015/03 was approved by the Ethical Committee for the Use of Experimental Animals (CEUA) of the Universidade Federal de Ouro Preto, Ouro Preto, MG, Brazil. All the experiments were performed to minimize animal suffering.

### 2.2. Epitope Mapping and Chimeric Proteins Design

The chimeric proteins used in this study were designed on the basis of epitopes mapped along *Leishmania infantum* proteins already described in the literature as vaccine candidates (Table 1). For epitope mapping, an integrative immunoinformatics approach described by [9,11] was used. The epitopes for T cells were selected on the basis of the highest scores provided by the predictive algorithms. We used a combination of NetCTL and NetMHC to predict MHC class I epitopes and NetMHCII for MHC class II epitopes, thus promiscuous epitopes were selected on the basis of their affinity towards human and mouse MHC alleles that are available for those algorithms. The algorithm NetCTL version 1.2 makes predictions of peptide–MHC class I binding and proteasomal C terminal cleavage, both using artificial neural networks, and transporter associated with antigen processing (TAP) transport efficiency using weight matrix. The tree predictions are then integrated [12]. The thresholds used were 0.15 for the weight on C terminal cleavage, 0.05 for weight on TAP transport efficiency, and 1.0 for epitope identification. NetMHC version 3.0. predicts binding of peptides to different HLA alleles using artificial neural networks and weight matrices, and the prediction is based on IC_50_ values where strong binder epitopes have an IC_50_ below 50 nM, and weak binder epitopes have an IC_50_ value below 500 nM [13]. NetMHCII version 1.0 was used, which predicts binding of peptides to 14 different HLA-DR alleles using position specific weight matrices (PSSM) [14]. The thresholds are based on IC_50_—50 nM for strong binder epitopes, and 500 nM for weak binder threshold score.

To compose the chimeric vaccines, these epitopes were repeated in tandem to enhance the immune response after immunization. Chimeric protein A has epitopes from histone protein (H2A), acid ribosomal protein P2 (LiP2a), acid ribosomal protein P0 (LiP0), *Leishmania* homologue of activated C kinase (LACK), and cysteine peptidase C (CPC). Chimeric protein B is composed of epitopes of cysteine peptidase proteins A and B (CPA and CPB), surface antigenic protein (PSA-50S), and specific amastigote protein A2 (A2). The linker GPGPG amino acid sequence was used to link the epitopes and increase protein stability and processing [15] (Figure S1).

### 2.3. Prediction of Epitope Conservancy among Leishmania Species

The proteome of *L. donovani* (BPK282A1 strain), *Leishmania major* (MHOM/IL/81/Friedlin strain), and *Leishmania braziliensis* (MHOM/BR/1966/M2903 strain) were retrieved from The Kinetoplastid Genomics Resource (TritrypDB). The proteome of *Leishmania amazonensis* (MHOM/BR/71973/M2269 strain) was retrieved from [16]. To evaluate the conservancy of the selected epitopes among these *Leishmania* species, the Immune epitope database (IEDB) epitope conservancy tool (http://tools.immuneepitope.org/tools/conservancy/) was used.

### 2.4. Prediction of Antigenicity, Allergenicity, and Physicochemical Properties of the Two Chimeras 

To predict the antigenicity of the two constructs (chimeras A and B), ANTIGENpro was used. This algorithm is based on sequences, pathogen-independent prediction method, and an alignment-free prediction method. For the allergenicity prediction, we used AlgPred, which uses a combination of predictive algorithms as described by [17]. The server uses several algorithms (SVMc + MAST + IgEepitope + ARPs BLAST) to check allergenicity of a query sequence, the prediction of allergens is based on similarity of known epitope with any region of a given protein. The server results are reliable and have 85% precision at −0.4 threshold.

To define the physical and chemical parameters associated with the constructs, ProtParam web server (from EXPASY) was used to define various physicochemical properties. Parameters as molecular weight (kDa), theoretical isoelectric point (pI), estimated half-life, and grand average of hydropathy were assessed.

### 2.5. Tertiary Structure Prediction and Refinement of the Models

The 3D structures of predicted chimera constructs were assessed by utilizing RaptorX [18], a web server for structure prediction. To improve the quality of the templates, a refinement of the tertiary structures was performed using GalaxyRefine web server [19]. The validation of refined models was carried out using ProSA [20], a web server. This server can predict the quality of the model which is indicated in the form of z-score. This score must be in the range of the characteristic for native proteins, otherwise it can indicate an erroneous structure. After that, Ramachandran plots were generated using RAMPAGE server [21] to determine the overall quality of predicted and refined models.

### 2.6. Molecular Docking to Highlight the Binding Affinity between the Chimeras and Mouse and Human TLR Receptors

For molecular docking, we used ClusPro2.0, which utilizes the Fourier correlation algorithm, filtering out the models with the amalgamation of desolvation and electrostatic energies. On the basis of the lowest binding energy, docked complexes were selected for further analysis [22]. The evaluation of the binding affinity between the refined constructs (chimeras A and B) and mouse and human toll-like receptors (TLRs) 3 and 4 was performed. The tertiary structures of TLR3 and TLR4 were retrieved from the PDB database [23,24]—for TLR3 we used id:3CIG (mouse) and id:2A0Z (human). Regarding TLR4, id:5IJB and id:4G8A were used, corresponding to mouse and human, respectively.

### 2.7. Chimera Proteins Synthesis 

The proteins (chimeras A and B) were synthetized by GenScript company; the genes were constructed, cloned, and expressed in prokaryote system (*Escherichia coli*) according to the manufacturer. The purification was performed by nickel column followed by characterization using SDS-PAGE and Western blot (Figure S2). The purified proteins were endotoxin free (<1 EU/μg) and with purity higher than 90% by SDS-PAGE. The endotoxins were measured according to the Food and Drug Administration FDA-approved techniques for endotoxin detection, therefore Genscript used Limulus amoebocyte lysate (LAL) assay. The specific LAL kinetic turbidimetric form was performed, which can detect down to 0.01 EU/mL.

### 2.8. Parasites

Promastigotes of *L. infantum*, strain OP46 (MCAN/*BR*/2008/*OP46*), maintained by passage in Syrian golden hamsters were cultured at 22–24 °C in medium LIT (liver infusion tryptose) with 100 U of penicillin G sodium and 100 μg of streptomycin sulfate per milliliter, and were sub-cultured in the same medium at an average density of 1 × 10^8^ cells/ml as described by [25]. The parasites were used for soluble *Leishmania* antigen (SLA) preparation, as described by [26], as well as for mice experimental challenge.

### 2.9. Immunization Regimens and Challenge of the Mice

For vaccine efficacy studies, eight 6- to 8-week-old female BALB/c mice per group (Centro de Ciência Animal (CCA) facility) were randomized by corporal weight and immunized three times biweekly subcutaneously in the back with 100 µl of vaccine formulations per mouse. The experimental groups were divided in SAL (animals that received sterile saline, 0.9% NaCl, pH 7.2–7.4), SAP (animals inoculated with 60 μg of saponin), ChiA+SAP (animals that received 10 μg of the chimeric protein A associated with 60 μg of saponin), and ChiB+SAP (animals inoculated with 10 μg of chimeric protein B associated with 60 μg of saponin). The doses were stablished on the basis of certain studies using chimeric proteins where 10 µg of the chimeric proteins per dose was in the average [27,28]. Mice were challenged by injecting 1 × 10^7^ of stationary *L. infantum* strain OP46 promastigotes intravenously, 15 days after the last immunization. 

### 2.10. Analyses of Polyfunctional T cell Phenotypes

Fourteen days after the last immunization, polyfunctional T cell phenotypes were assessed in splenocytes of mice. The evaluation was performed before infection because we wanted to analyze the vaccine immune responses triggered in BALB/c mice in more detail, and because the identification of these poly-functional T cells is more evident after the last immunization but before the infection [6,29]. Cell suspensions were incubated in RPMI supplemented with 1% L-glutamine and 10% fetal bovine serum and plated in 96-well round-bottom (Costar, USA) culture plates at a concentration of 5 × 10^5^ cells per well. Cells were cultured for 24 h at 37 °C with 5% CO_2_ in the presence of SLA (50 μg/ml). Brefeldin A (SIGMA) was added (10 μg/ml) at 20 h of incubation. Afterwards, cells were blocked with anti-mouse CD16/CD32 (Mouse BD FC block, 0,5 μg/well) harvested, washed, treated with Phosphate buffer saline (PBS) plus an inert protein (serum albumin 5%), and stained with anti-mouse CD3 FITC (clone 145.2C11), anti-mouse CD4 BV605 (clone RM4-5), anti-mouse CD8α BV786 (clone 53-6.7), anti-mouse CD62L BV510 (clone MEL-14), and anti-mouse CD44 APC (clone IM7) (BD Biosciences Bioscience, USA) at room temperature for 30 minutes. Cells were fixed with FACS fixing solution (10 g/L paraformaldehyde, 10.2 g/L sodium cacodylate, and 6.63 g/L sodium chloride, pH 7.2), washed, and treated with PBS buffer containing 0.5% saponin for permeabilization. Cells were stained with anti-mouse IFN-γ AF700 (clone XMG1.2), anti-mouse tumor necrosis factor (TNF-α )PE-Cy7 (clone LG.3A10), and anti-mouse IL-2 PE (clone JES6-5H4) (BD Biosciences Bioscience, San Jose, CA, USA). Cells were acquired (300,000 events) on LSR Fortessa cytometer (BD Biosciences, USA) using FACSDiva software. For analysis in FlowJo software, dead cells were excluded after FVS450 stain and alive cells were gated for CD4^+^ and CD8^+^ T cells and intracellular cytokine production. Polyfunctional cells were analyzed through the Boolean gate strategy.

### 2.11. Proliferation Assay and Intracellular Cytokine Stain 

The proliferation of antigen experienced T cells was assessed by Carboxyfluorescein diacetate succinimidyl ester (CFSE) assay, 30 days after mice challenge, as described by [11]. Briefly, spleen’s cell suspensions were incubated in 5 μM CFDA-SE and the labeled cells were washed thoroughly before plating in 96-well round-bottom culture plates at a concentration of 5 × 10^5^ cells per well. Cells were cultured for 5 days at 37 °C with 5% CO_2_ in the presence of SLA (50 μg/ml). After incubation, cells were treated with 10 μg/ml of brefeldin A (Sigma) for 4 h. Afterwards, cells were blocked with anti-mouse CD16/CD32 (0.5 μg/well) and harvested, washed, treated with PBS and an inert protein (serum albumin 5%), and stained with anti-mouse CD3 BV650 (clone 145.2C11), anti-mouse CD4 BV605 (clone RM4-5), and anti-mouse CD8α BV786 (clone 53-6.7) (BD Biosciences Bioscience, USA) at room temperature for 30 minutes. Cells were fixed with FACS fixing solution (10 g/L paraformaldehyde, 10.2 g/L sodium cacodylate, and 6.63 g/L sodium chloride, pH 7.2), washed, and permeabilized. Cells were stained with anti-mouse IFN-γ AF700 (clone XMG1.2), anti-mouse TNF-α PE-Cy7 (clone LG.3A10), and anti-mouse IL-2 PE (clone JES6-5H4) (BD Biosciences Bioscience, USA). Cells were acquired (300,000 events) on LSR Fortessa cytometer (BD Biosciences, USA) using FACSDiva software. For analysis in FlowJo software, dead cells were excluded after FVS780 stain and alive cells were gated for CFSE-stained CD4^+^ and CD8^+^ T cells and intracellular cytokine production.

### 2.12. Analyses of Memory T cell Phenotypes 

Central and effector memory T cells were analyzed 30 days post-challenge, as described by [11]. Splenocytes from animals were plated at 5 × 10^5^ cells per well in duplicate in 96-well round-bottom plates. Cells were treated with the same conditions described above. After 5 days of culture, cells were then prepared for flow cytometry analysis. Samples were blocked with anti-mouse CD16/CD32 (0.5 μg/well) and stained with surface markers at room temperature using the following antibodies: anti-mouse CD3 FITC (clone 17A2), anti-mouse CD4 BV605 (clone RM4-5), anti-mouse CD8α PerCP-Cy5.5 (clone 53-6.7), anti-mouse CD44 APC (clone IM7), anti-mouse CD45RA BV711 (clone 14.8), anti-mouse CD62L AF700 (clone MEL-14), anti-mouse CD127 BV510 (clone SB/199), and anti-mouse CD197 BV421 (clone 4B12). The events were acquired (300,000 cells) on an LSR Fortessa cytometer (BD Biosciences) using FACSDiva software. For analysis, dead cells were excluded after FVS780 stain and live cells were used for further analyses.

### 2.13. Evaluation of the Parasite Burden in the Spleen by Quantitative PCR

Parasite burden in the spleen was analyzed 30 days after infection by real-time PCR (qPCR) quantification, as described by [30]. Briefly, at the end of the study, the mice were euthanized, and the spleens were extracted and used for genomic DNA isolation with the CTAB reagent. The *L. infantum* spleen burden was assessed by PCR quantification of DNA polymerase (forward primer: 5′-TGTCGCTTGCAGACCAGATG-3′; reverse primer: 5′-GCATCGCAGGTGTGAGCAC-3′; Taqman probe: VIC 5′-AGGAAACCTGTGGAGCC-3′ MGB NFQ). The amounts of mouse chromosomal DNA in extracted spleen were analyzed using murine TNF-α primers (5- TCCCTCTCATCAGTTCTATGGCCCA-3; 5-CAGCAAGCATCTATGCACTTAGACCCC-3) that amplify a 170 bp product. The PCR reactions were conducted as follows: one incubation step at 50 °C for 2 min and an initial denaturation step at 95 °C for 10 min, followed by 40 cycles of denaturation at 95 °C for 15 s and annealing-extension at 60 °C for 1 min. All samples were run on MicroAmp optical 96-well reaction plates (Applied Biosystems) sealed with MicroAmp optical adhesive film (Applied Biosystems). Each 96-well reaction plate contained a standard curve in triplicate (efficiency, 96.0%; *r^2^* = 0.99) in duplicate samples. The result was expressed as number of amastigotes DNA copies per spleen of mice.

### 2.14. Statistical Analysis

The data were analyzed through Graph Pad Prism 8.0 software and expressed as mean plus/minus standard deviation (SD). First, the normality of the data was assessed using the Shapiro–Wilk test and statistical differences were analyzed by one-way ANOVA and Kruskal–Wallis test followed by Dunnett’s and Dunn’s multiple comparison tests, respectively. Correlation analyses were performed by Pearson’s *r* test. Differences were considered significant when *p-*value <0.05.

## 3. Results

### 3.1. In Silico Construction of the Chimeras A and B

Herein, a total of 12 epitopes (ligands for mouse and human MHC class I and II alleles) were predicted to construct chimera A and chimera B. These epitopes were mapped in the sequence of nine proteins described in the literature as potential vaccine candidates for VL. The peptides are represented in Table 1, where it can be seen that most of them were highly conserved across *L. donovani* and *L. major* species. To construct the chimeras, the epitopes were repeated in tandem to enhance the immunogenicity, and GPGPG sequence was used as a linker. The final constructions of the chimeras were predicted to be highly antigenic (predicted probability of antigenicity chimera A = 0.896793 and chimera B = 0.790044), and they were also revealed to be nonallergic in nature. Prediction by SVM method based on amino acid composition (chimera A score = −0.96747231 and threshold = −0.4; chimera B score = −0.55381796, threshold = −0.4). Prediction was based on the SVM method which was based on dipeptide composition (chimera A score = 0.27467095, threshold = −0.2; chimera B score = 0.14230073 and threshold = −0.2). For predictions based on BLAST analyses, no hits were found for both chimera sequences and they did not contain experimentally proven Immunoglobulin E (IgE) epitope.

Regarding physicochemical properties, chimera A was found to have 40 kDa of molecular weight and Chimera B has 38 kDa. The theoretical isoeletric points (pI) for the chimeras A and B were found to be 9.8 and 9.57, respectively, showing a basic nature. For chimeras A and B, an in silico estimated half-life of 30 h in mammalian reticulocytes and 20 and 10 h in yeast and *E. coli,* respectively, was observed. Both the chimeras are hydrophilic in nature according to grand average of hydropathicity (GRAVY).

### 3.2. Tertiary Structure Prediction and Binding Affinity between the Chimeras and Mouse and Human TLR Receptors

**Figure 1:**
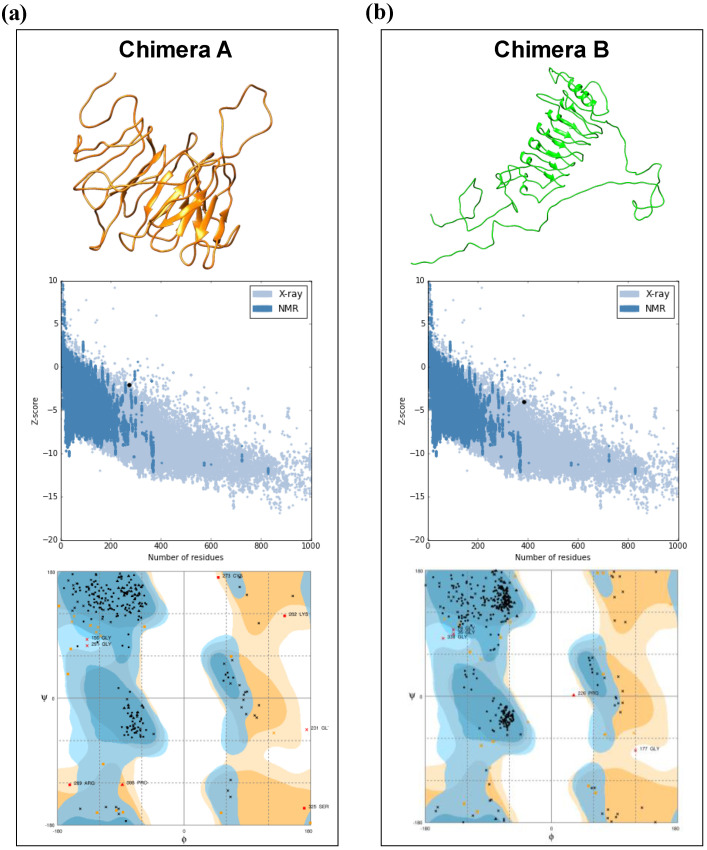
Illustrative representation of 3D structures for chimeras A and B and the validation parameters provided by Rampage and ProSA web servers. Chimera A (**a**) is represented by orange color and chimera B (**b**) is represented by green color. ProSA analysis and Ramachandran diagrams are represented in the figure for both chimeras (above 3D structures). The quality of the models is given by the z-score (−3.05) predicted by the ProSA, whereas Ramachandran diagrams show the distribution of the amino acid in favored, allowed, and disallowed regions.

RaptorX webserver was used to predict the tertiary structure and three-dimensional (3D) model of the chimera proteins. Further, the refinement of the chimeras’ models was assessed using Galaxy Refine tool. To validate the 3D models, RAMPAGE and ProSA were used, and the initial model of the chimeras A and B showed 85.4% and 88.5% of residues, respectively, in the favored regions, whereas after refinement, the models reached to 90.1% and 92.7%, respectively. The z-score returned for chimera A was -2.02 and for chimera B it was -4.01, and this showed that the quality of the predicted model is acceptable (Figure 1a,b).

**Figure 2:**
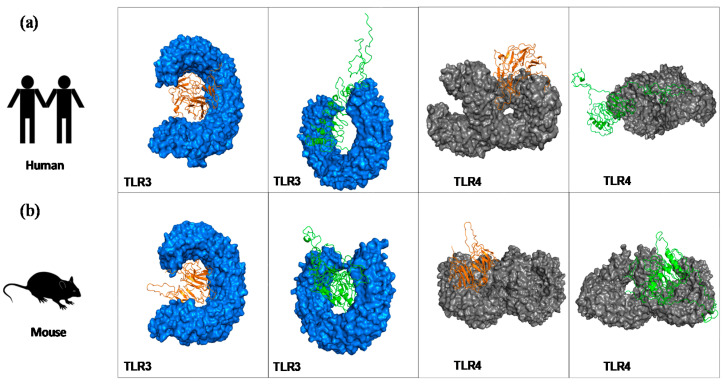
Interaction of refined models for chimeras A and B with toll-like receptors using molecular docking (ClusPro2.0 web server)**.** Representation of the best docked-complex between chimeras and human (**a**) and mouse (**b**) toll-like receptor (TLR)3 (blue) and TLR4 (grey). Chimera A (a) is represented by orange, chimera B is represented by green.

After the molecular docking of chimera proteins and human (Figure 2a) and mice (Figure 2b) TLR3/TLR4 receptors, several models were generated for the eight complexes. Among them, we selected only models that presented the lowest energy and where the receptors were properly occupied by the constructs; thus, Figure 2 shows the best-docked complexes. Toll-like receptors (TLRs) are a group of receptors that can recognize pathogen-associated molecular patterns (PAMPs), and they play a crucial role in modulating immune response. TLR4 and TLR3 are expressed on cell membrane surface and on endosomes, respectively. Their activation results into functioning of intracellular signalling pathway of nuclear factor kappa-B (NF-κB) and cytokine production, leading to innate immune system activation and ultimately long-lasting adaptive immunity that is important against various pathogens [31]. Thus, we wanted to measure in silico the capacity of the chimeras to bind those TLRs, suggesting that vaccines should have the capacity to enter into immune cells and be able to trigger an immune response.

### 3.3. Chimeras A and B Designed by Immunoinformatics Tools Elicited a Different Pattern of Polyfunctional T Cells in the Spleen of Immunized Mice

**Figure 3:**
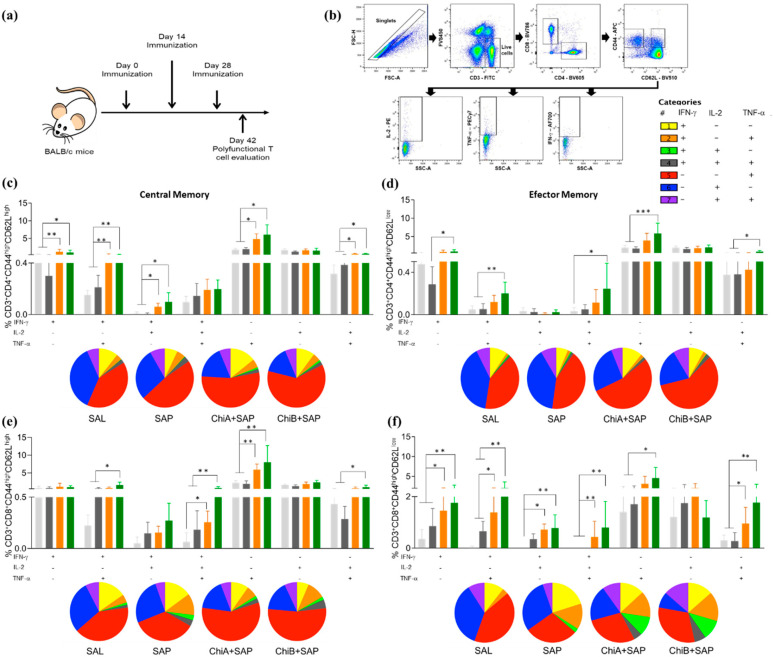
Percentage of polyfunctional T cells in spleens of immunized mice with ChiA+SAP (animals that received 10 μg of the chimeric protein A associated with 60 μg of saponin) and ChiB+SAP (animals inoculated with 10 μg of chimeric protein B associated with 60 μg of saponin). Polyfunctional T cells were evaluated by percentage of intracellular IL-2, TNF-α, and IFN-γ producing T cells (central and effector memory) at the same time. (**a**) BALB/c mice were immunized three times and polyfunctional T cells were assessed in spleen 14 days after the last immunization using multicolor flow cytometry. (**b**) Representative plot of the gating strategy to characterize the multifunctional T cell producers of intracellular IFN-γ, TNF-α, and IL-2 using the Boolean gate strategy. (**c**) The percentage of CD3^+^CD4^+^CD44^high^CD62L^high^ and (**d**) CD3^+^CD4^+^CD44^high^CD62L^low^ cells producing single, double, and triple cytokines after in vitro stimulation with SLA (soluble *Leishmania* antigen). (**e**) The percentage of CD3^+^CD8^+^CD44^high^CD62L^high^ and (**f**) CD3^+^CD8^+^CD44^high^CD62L^low^ producing single, double, and triple cytokines in vitro after stimulation with SLA. The groups SAL (animals that received sterile saline, 0.9% NaCl, pH 7.2–7.4), SAP (animals inoculated with 60 μg of saponin), ChiA+SAP, and ChiB+SAP are represented by the colors light grey, dark grey, orange, and green, respectively, and pizza graphs represent the pattern of cytokine production. Data are expressed as means plus/minus standard deviation of two independent experiments (*n* = 8). Significant differences between the groups are represented by *p-*values: * *p*-value < 0.05, ** *p*-value < 0.005, *** *p*-value < 0.0005.

In this step, BALB/c mice were immunized (2 weeks of interval) with the chimeras, and the animals were sacrificed 14 days after the last dose (Figure 3a). To determine the capacity of the chimeras to elicit and develop polyfunctional central and effector memory cells, single, double, and triple intracellular cytokine productions were evaluated in T cells using multicolor flow cytometry. The gating strategy is shown in Figure 3b. The analysis of single-cell cytokine production indicated that IFN-γ and TNF-α produced by central memory (CM) CD4^+^ T cells (Figure 3c) were significantly higher in the ChiA+SAP and ChiB+SAP groups when compared to the control groups (SAL, SAP). There was no difference for IL-2 single production between the groups. On the other hand, the double cytokine production (IL-2 + IFN-γ IL-2 + TNF-α and IFN-γ + TNF-α) was higher in the vaccinated groups comparing to SAL and SAP, although there were no statistical differences in the triple cytokine production in the immunized groups. For effector memory (EM) CD4^+^ T cells (Figure 3d), ChiB+SAP was able to induce higher single (IFN-γ and TNF-α), double (IL-2 + TNF-α and IFN-γ + TNF-α), and triple (IFN-γ + IL-2 + TNF-α) cytokine production when compared to SAL and SAP. Regarding the subpopulation of CM CD8^+^ T cells (Figure 3e), it a higher production of triple cytokine in the cells of the immunized mice (ChiA+SAP and ChiB+SAP) was observed when compared to SAL and ChiB+SAP-immunized mice in comparison to SAP group. In addition, ChiB+SAP promoted an enhancement in the percentage of CM CD8^+^ producing double cytokines (IL-2 + TNF-α and IFN-γ + TNF-α) when compared to the control groups. Changes in the patterns of cytokine production were found in EM CD8^+^ T cells (Figure 3f), where ChiB+SAP induced higher frequency of cells producing single (except IL-2), double, and triple cytokines when compared to SAL and SAP. Taken together, these data show that the chimeric vaccines constructed through immunoinformatics induced an immunogenicity and increased the polyfunctionality central and effector T cell subpopulations.

### 3.4. Chimeras a and B Not Only Induced Proliferation of T Cells but Also Enhanced the Production of Intracellular Cytokines by These Cells in the Spleen of Immunized Mice and after Being Challenged with L. Infantum

**Figure 4:**
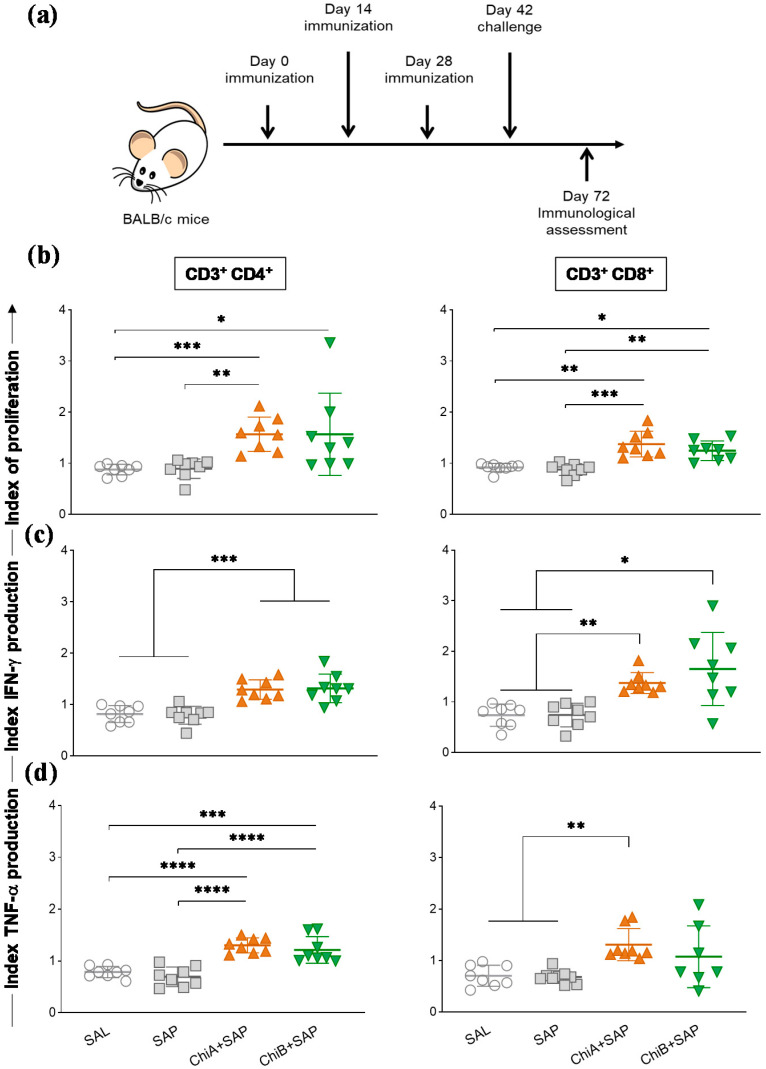
Splenic immune response after mice immunization with chimeric vaccines and challenge with *L. infantum*. (**a**) BALB/c mice were immunized three times and challenged with *L. infantum* promastigotes. Four weeks later, splenocytes were obtained and proliferation and intracellular cytokine production (IFN-γ and TNF-α) were assessed through flow cytometry. (**b**) Graphs represent the index of T cell proliferation (culture index stimulated by the control culture (SC/CC) ratio) of T-CD4^+^ and T-CD8^+^ cells after in vitro stimulation with SLA (soluble *Leishmania* antigen). (**c**) Representation of IFN-γ and (**d**) TNF-α production indexes (SC/CC ratio) of T-CD4^+^ and T-CD8^+^ cells after in vitro stimulation with SLA. Indexes were calculated on the basis of SLA-stimulated (SC) cultures divided by the control culture (CC). Data are expressed as means ± SD of two independent experiments (*n* = 8). The *p*-values represent the difference between the groups: * *p-*value *<* 0.05, ** *p-*value *<* 0.005, *** *p-*value *<* 0.0005, and **** *p-*value *<* 0.0001.

To evaluate the efficiency of the chimeras A and B to promote specific immune responses in vivo, BALB/c mice were immunized with three doses of chimeras with saponin (2-week intervals) and then challenged with promastigotas of *L. infantum*. Thirty days after challenge, they were euthanized (Figure 4a). The results of the proliferation of splenocyte and intracytoplasmic cytokine production were expressed as culture index stimulated by the control culture (SC/CC). The representative plot of the gating strategy for CFSE-labelled cells is shown in Figure S3. According to the data (Figure 4b), ChiA+SAP induced a higher CD4^+^ and CD8^+^ T cell proliferation when compared to SAL and SAP groups, whereas ChiB+SAP induced a significant CD4^+^ T cell proliferation compared to SAL. Regarding intracellular IFN-γ production by CD4^+^ and CD8^+^ T lymphocytes, we observed a significant increase in ChiA+SAP and ChiB+SAP compared to SAL and SAP groups (Figure 4c). Further, ChiA+SAP and ChiB+SAP were capable of increasing the production of TNF-α by CD4^+^ T cells and only ChiA+SAP increased the production of this cytokine by CD8^+^ T cells when compared to control groups (Figure 4d).

### 3.5. Chimeras A and B Promoted a Strong Reduction in Parasite Burden and Developed Central and Effector Memory of T Cells in the Spleen of Immunized and Challenged Mice

**Figure 5:**
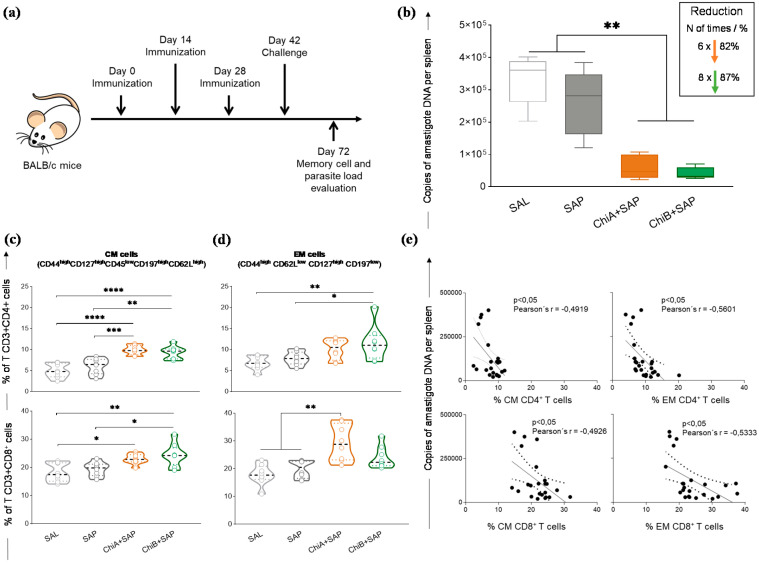
Parasite burden and generation of memory cells in the spleen of immunized mice with chimeric vaccines and challenged with *L. infantum*. (**a**) BALB/c mice were immunized three times and challenged with *L. infantum* promastigotes. Four weeks later, they were sacrificed for further analyses. (**b**) Parasite burden was assessed in the spleen using quantitative PCR and results are expressed in amastigote DNA copies per spleen. (**c**) Violin plots represent the percentage of central memory T cells (CD3^+^ CD4^+^ or CD8^+^ CD127^high^ CD44^high^ CD45RA^low^ CD62L^high^ C197^high^) and (**d**) effector memory T cells (CD3^+^ CD4^+^ or CD8^+^ CD62L^low^ CD44^high^ CD127^high^ C197^low^) in mice splenocytes after in vitro stimulation with SLA. (**e**) Correlation analyses between parasite load and frequency of T CD4^+^ and CD8^+^ central memory (CM) and effector memory (EM) cells are shown by Pearson´s *r*. Data on the graphs are represented as means ± SD of two independent experiments (*n* = 8) and *p*-values represent the difference between the groups: * *p-*value *<* 0.05, ** *p-*value *<* 0.005, *** *p-*value *<* 0.0005, and **** *p-*value *<* 0.0001.

We determined the capacity of these chimeric vaccines to protect immunized mice. For that, after the immunization, BALB/c mice were challenged and sacrificed after 30 days (Figure 5a). Total spleen DNA was used for the quantification of parasite burden. The results are shown in Figure 5b, where it can be seen that both chimeric vaccines promoted a significant reduction in parasite burden when compared to SAL and SAP groups. In this sense, this reduction was approximately 82% and 87% for ChiA+SAP and ChiB+SAP, respectively, when compared to the SAL group.

Furthermore, to characterize memory phenotypes of T cells, we investigated CM and EM cells in the splenocyte cultures using a multicolor panel. CM T cell phenotypes were characterized by CD127^high^ CD44^high^ CD45^low^ CD62L^high^ CD197^high^ and EM cells were characterized by CD62L^low^ CD44^high^ CD127^high^ CD197^low^. The results demonstrated that, after challenge, the percentage of CM CD4^+^ T-cells was higher in ChiA+SAP and ChiB+SAP groups when compared to control groups (Figure 5c). Regarding the subpopulation CD8^+^, it was observed that ChiA+SAP and ChiB+SAP enhanced the percentage of CM cells compared to SAL and ChiB+SAP, which also enhanced these cells when compared to SAP. Concerning EM cells, we found a high percentage of CD4^+^ T cells only in ChiB+SAP compared to SAL and SAP. On the other hand, for CD8^+^ T cells, only ChiA+SAP was able to generate these memory cells when compared to the control groups (Figure 5c). Moreover, we observed a negative correlation (Figure 5d) between the parasite burden and the percentage of CD4^+^ CM cells in the spleen (Pearson’s *r* = −0.4919; *p-*value <0.05). In addition, we found the same negative correlation for CD8^+^ CM cells (Pearson’s *r* = −0.4926, *p-*value <0.05), CD4^+^ EM cells (Pearson’s *r* = −0.5601, *p-*value <0.05), and CD8^+^ EM cells (Pearson’s *r* = −0.5333, *p-*value <0.05) (Figure 5e).

## 4. Discussion

The use of immunoinformatics capable of predicting immunodominant epitopes has been used in numerous studies. This methodology has already been described as being highly accurate for mapping immunogenic epitopes to *Leishmania* proteins [2,9,11,32]. Therefore, immunoinformatics can result in a huge gain for the design of multiepitope vaccines against neglected diseases, in this case, visceral leishmaniasis. 

In this context, the present study focused on the design of multi-epitope chimeric vaccines that can afford a robust level of protective immunity against *L. infantum*. For that, we mapped potential T cell epitopes on nine known proteins that have immunogenic capabilities that have been described in the literature. Therefore, according to the computational methodology proposed by [9], we mapped the highest score epitopes provided by immunoinformatics concerning the histone H2A, Lip2a, Lip0, LACK, and CPC (chimera A) proteins, as well as CPA, CPB, PSA-50S, and A2 proteins (chimera B) (Figure S1). Thus, we could observe that the components chosen to construct both chimera A and chimera B are antigens that stimulate lymphocyte proliferation and IFN-γ production. Further, the profile of cells stimulated with these antigens may also have a balanced Th1/Th2 polarization. This is observed by induction of humoral response with the production of immunoglobulins, but at the same time the antigens are able to trigger IFN-γ production and in vitro cell proliferation of mouse splenocytes [33,34,35,36,37,38,39,40].

The final construct for chimeras A and B has epitopes with high score for the algorithms used, conservancy across *L. donovani* and *L. major*, and highly antigenic and non-allergic for dogs and/or human use. Their predicted physicochemical features demonstrated that they are stable, hydrophilic (negative values for GRAVY), and basic nature. The 3D structures were validated using Ramachandran plot and ProSA webserver, indicating that the quality of the models is satisfactory [41,42,43,44,45]. Various studies have been shown that chimera constructs that have an affinity to toll-like receptors (mainly TLR-3, TLR-4, and TLR-9) may have the ability to enhance the immune response against intracellular pathogens [42,43,44,45,46]. In this sense, we performed molecular docking showing the interaction between chimeras A and B with mouse and human TLR-3 and TLR-4. For the conformational stability of docked complexes, energy minimization was achieved to minimize the potential energy of the complete systems [44]. In summary, both evaluated chimeras interacted with mice and human TLR-3 and TLR-4 demonstrating, the *in silico* capacity to be immunogenic. The cloning and expression of the chimeras was well succeeded using *E. coli* system, as shown in Figure S2.

A critical feature of successful vaccines against *Leishmania* spp. is their ability to induce multifunctionality regarding cytokine production and immunological memory [5,6,47]. Therefore, we chose IFN-γ, TNF-α, and IL-2, focusing on the objective of performing the multifunctional study to qualify both central and effector memory T cells. Some studies have already shown that multifunctional T cells that produce these cytokines are more effective when compared to individual production for parasite elimination and infection control [5,6,10,48]. Thus, our data reinforce that chimeras A and B associated with the adjuvant have this feature to change the patterns of cytokine production. These vaccines can elicit multifunctional CD4^+^ and CD8^+^ of CM and EM T cells, which are crucial for the elimination of the parasite [5,6,10,48]. Additionally, the Chimeric vaccines trigged T lymphocyte activation through the proliferation of CD4^+^ and CD8^+^ subpopulations. The results of T lymphocyte proliferation in the vaccine groups after *L. infantum* stimulation further support the findings that IFN-γ is released after this activation, and we can observe the generation of long-term immunological memory against *Leishmania* spp. [10,49,50,51,52]. Gamma interferon (IFN-γ, a cytokine considered central for protection against *Leishmania* spp.) is evaluated and investigated in practically all studies involving vaccines against leishmaniasis. This cytokine is critical for monitoring, screening, and designing effective vaccines against these intracellular protozoa [4,10,32,53,54]. Another effector cytokine is TNF-α, which plays a crucial role in the control of intracellular protozoan [55,56]. Our data support the role of these cytokines to orientate a type 1 response that is paramount to protection against *Leishmania* spp. [6,57]. Moreover, our results suggest that the protection conferred by the chimeric multiepitope protein could be associated with IFN-γ production by both T lymphocyte subpopulations.

To date, several vaccine candidates against the different types of leishmaniasis have been proposed and with promising results in murine models, but none of them have achieved the market for human immunization and VL prevention. This may be due to issues related to the different levels of immune memory generation that are currently agreed upon the guidelines for vaccine development in different research groups [6,52,58,59]. In our study, we characterized memory T cell phenotype in the spleen of the immunized animals. The results showed that both chimeric vaccines were able to trigger central memory in both CD4^+^ T lymphocytes and CD8^+^ T lymphocytes. Some studies have shown that these cells play a fundamental role in the immune response, being responsible for the renewal of immune system memory cells [60]. There is a compartmentalization of these memory cells, as demonstrated in studies in which central memory cells differentiate and expand in the spleen and may recirculate into secondary lymphoid organs [5]. Regarding the findings of effector memory cells, only ChiB+SAP was able to generate CD4^+^ EM T cells, and ChiA+SAP promoted an increase in CD8^+^ EM T cells. Effector memory cells have the important capacity to migrate to the sites of infection [61]. Thus, the chimeric vaccines tested in this study were able to generate effector memory lymphocytes in the splenic compartment accompanied by a decrease in parasitic load on the organ.

It is difficult to point out which profile, central or effector memory, would be responsible for protection in experimental models. This is due to the heterogeneity of responses generated in leishmaniasis immunoprophylaxis studies in different species or animal models, in which there is no consensus on which memory T cell profile would characterize the vaccine candidate as ideal or not [6]. Thus, it is believed that both profiles add a lot to this process of parasite control and elimination [29,50,59]. Our results corroborate with these studies, as we found negative correlation between the percentage of CM and EM T cells and parasite burden in the spleen. This shows that the augment of memory cells probably leads to parasite control and their elimination in the organs. In the splenic compartment there is the establishment of a chronic infection that occurs later and is not self-resolving as observed in the liver, which makes this organ one of the main targets in studies evaluating the efficacy of vaccines in BALB/c model [62]. Given the above information, our findings demonstrated an important parasite reduction in the spleen of vaccine groups of 82% and 87%, respectively, for ChiA+SAP and ChiB+SAP. 

Few studies evaluating polyepitope chimeric vaccines have been reported in the literature. Within the context of VL, there is a restricted number of studies on chimeric vaccines. Regarding challenges with *L. infantum*, parasite burden results in the spleen obtained by us were superior when compared to the 42% load reduction after 10 weeks of infection in a study conducted by [63]. Regarding the *L. donovani* challenge, the authors of [4] observed a surprising 91% reduction in parasite burden on the same organ after 21 days of infection. It is interesting to note that in this study we used 10 µg per dose of each of the chimeras, much smaller amounts compared to other studies that used 20, 25, 100, or even 200 µg of multiepitope chimeric protein [10,63], indicating the higher capacity of our chimeras in generating immunogenicity, which must be directly associated with parasite control. We highlight the possibility of missing or omitting potential epitopes due to in silico prediction as a possible disadvantage noted in studies employing immunoinformatics. On the other hand, this methodology allows large-scale screening, leading to the mapping of important epitopes over a short period of time, which can potentially be very advantageous when used for leishmaniasis vaccines [3]. 

## 5. Conclusions

Taking all these findings together, this study is promising in the field of development of multiepitope chimeric vaccines for VL, being rationally designed using immunoinformatics and employing different computational approaches. Thus, the vaccines elicited multifunctional T cells and induced immunogenicity with CD4^+^ and CD8^+^ T lymphocyte proliferation and IFN-γ and TNF-α production. Besides this, vaccines instigated the development of central memory and effector of T lymphocytes in mouse splenocytes, which led to a decrease in parasite load in splenic tissue. To achieve success in vaccine development for leishmaniasis, many rational approaches and new techniques for evaluation of polyfunctional and memory cells should be taken. In this sense, this study will contribute a large amount to the field of development of vaccines against VL, a critical neglected disease affecting thousands of people around the world. 

## 6. Patent

The patent of the chimeric vaccines used in this study was deposited under the register number BR10201800819 in the *Instituto Nacional da Propriedade Industrial* (INPI), Brazil.

## Figures and Tables

**Table 1:** MHC class I and II epitopes of known *Leishmania infantum* proteins that have been shown as candidate vaccines for leishmaniasis. The amino acid sequence, alleles, and conservancy of these promiscuous epitopes are represented in the table. The epitopes were selected through an immunoinformatics pipeline describe by [9,11] and they showed a high score for the algorithms NetMHC, NetCTL, and NetMHCII.

Chimera	Protein	Epitope Sequence	MHC Class I and II Alleles					Conservancy of the Selected Epitope across *Leishmania* Species (%)			
								*L. donovani*	*L. major*	*L. amazonensis*	*L. braziliensis*
**Chimera A**	**Histone protein (H2A)**	**KKRCRLNPR**	**H2-Dk**					100	89	100	66
		DDISSLLKNVTLSHS	HLA-DRB1*1302	HLA-DRB1*0101	HLA-DRB1*0401	HLA-DRB1*0404	HLA-DRB1*1501	100	87	100	66
	Acid ribosomal protein P2 (LiP2a)	AAKMSAMPAASSGAA	HLA-DRB1*0901	HLA-DRB1*0101				100	100	100	93
		MSTKYLAAY	HLA-A*01	HLA-A*26	HLA-B*62	HLA-A*03	HLA-B*58	100	100	100	100
	Acid ribosomal protein P0 (LiP0)	VDAFKNLLAVSVATSYEF	HLA-DRB1*0101	HLA-DRB1*0701	HLA-DRB1*1501	HLA-DRB1*1302		100	100	100	94
		AHRVKAPAR	H2-Dk					100	100	100	89
	Leishmania homologue of activated C kinase (LACK)	WSADGNTLY	HLA-A*01	HLA-A*26	HLA-B*62	HLA-B*58		100	100	100	100
		DRSIRMWDLRNGQCQ	HLA-DRB4*0101	HLA-DRB1*1501				100	100	100	93
		ATERSLSVY	HLA-A*01	HLA-A*26	HLA-B*62			100	100	100	78
	Cysteine peptidase C (CPC)	GYLVSGKSL	HLA-A*24	H2-Kd				100	100	100	78
		WTASADNGY	HLA-A*01	HLA-A*26	HLA-B*62	HLA-B*58		100	100	100	89
		LVKYKGGTSYSVKGE	HLA-DRB1*0101	HLA-DRB1*0701	HLA-DRB1*1501			100	100	100	60
**Chimera B**	Cysteine peptidase A (CPA)	MTEDYMGMY	HLA-A*01	HLA-A*26	B62			89	100	67	100
		AKRRRLPTT	H2-Dk					100	100	78	78
		RPDFMNMTPRGVPLE	HLA-DRB1*0101	HLA-DRB1*0701	HLA-DRB1*1501	HLA-DRB1*1302	HLA-DRB5*0101	100	87	93	67
	Cysteine peptidase B (CPB)	SKKFSHPSL	HLA-B*39					100	66	89	56
		AGALVMGTALLTESA	HLA-DRB1*0101	HLA-DRB1*0404	HLA-DRB1*1302			100	60	80	67
		RTDRQSCLY	HLA-A*01	HLA-B*58	HLA-A*03			100	78	89	78
	Surface antigenic protein (PSA-50S)	DSWSRLQGLTSLTLS	HLA-DRB1*0101	HLA-DRB1*1501				53	87	53	53
		LPPEWAAMP	H2-Dd					78	89	78	78
		LTDERTCLV	HLA-A*01	HLA-A*02				67	100	78	67
	Amastigote protein A2 (A2)	GKGLRAPPL	H2-Dk					100	100	78	56
		SQAGDVFAL	HLA-B*39	HLA-B*62	HLA-B*44	HLA-A*02	HLA-B*27	100	89	78	56
		GPHLRGGAVTSSVVT	HLA-DRB1*0101	HLA-DRB1*0701				100	87	67	60

## Supplementary Materials

The following are available online at https://www.mdpi.com/2076-393X/8/2/252/s1: Figure S1: Illustration of the Chimera’s constructions. The multi-epitope vaccine sequence consisting of 24 MHC class I and II ligands. GPGPG sequence was used as linker to join the epitopes. H2A = Histone protein 2, LiP2a = Acid ribosomal protein P2, LiP0 = Acid ribosomal protein P0, LACK = Leishmania homologue of activated C kinase, CPC = Cysteine peptidase C, CPA = Cysteine peptidase A, CPB = Cysteine peptidase B, PSA-50S = Surface antigenic protein, A2 = Amastigote protein A2, Figure S2: Evaluation of the expression of chimeric proteins A and B in the E. coli system. The expression was confirmed by SDS-PAGE as described in (a) and (b) for Chimeras A and B respectively. M1 = protein marker 1 (120 to 10 kDa); 1 = Bovine Serum Albumin (2 μg); 2 = 2 μg of chimeric protein A (~40 kDa) or B (~38 kDa). Also, the expression of the Chimera A (c) and Chimera B (d) was confirmed by Western Blot using anti-His antibody. M2 = protein marker 2 (120 to 22 kDa); 3 = 2 μg of chimeric protein A (c) and B (d), Figure S3: Representative plot of the gating strategy for CFSE-labelled cells to evaluate the proliferation of splenocytes. The first step was to select the single cells, then the live cells using (FVS780). To characterize the T-lymphocytes anti-CD3, anti-CD4 and anti-CD8 were used followed by the proliferation analyses selecting the CFSElow-labelled cells.
