# Supplementary material for: Chimeric Vaccines Designed by Immunoinformatics-Activated Polyfunctional and Memory T cells that Trigger Protection against Experimental Visceral Leishmaniasis
Source: Vaccines (Basel). 2020 May 27;8(2):252. doi: 10.3390/vaccines8020252 (PMC7349981; doi:10.3390/vaccines8020252)
Supplement: Supplementary file 1 [file vaccines-08-00252-s001.pdf]

Review

Covid-19 Pandemic: What Changes for Dentists and Oral Medicine Experts? A Narrative Review and Novel Approaches to Infection Containment

Maria Eleonora Bizzoca [1], Giuseppina Campisi [2] and Lorenzo Lo Muzio [1,3,*]

[1] Department of Clinical and Experimental Medicine, University of Foggia,71121 Foggia, Italy; marielebizzoca@gmail.com
[2] Department of Surgical, Oncological and Oral Sciences (Di.Chir.On.S.), University of Palermo, 90121 Palermo, Italy; campisi@odonto.unipa.it
[3] C.I.N.B.O. (Consorzio Interuniversitario Nazionale per la Bio-Oncologia), 66100 Chieti, Italy;
* Correspondence: lorenzo.lomuzio@unifg.it

Received: 3 May 2020; Accepted: 23 May 2020; Published: 27 May 2020

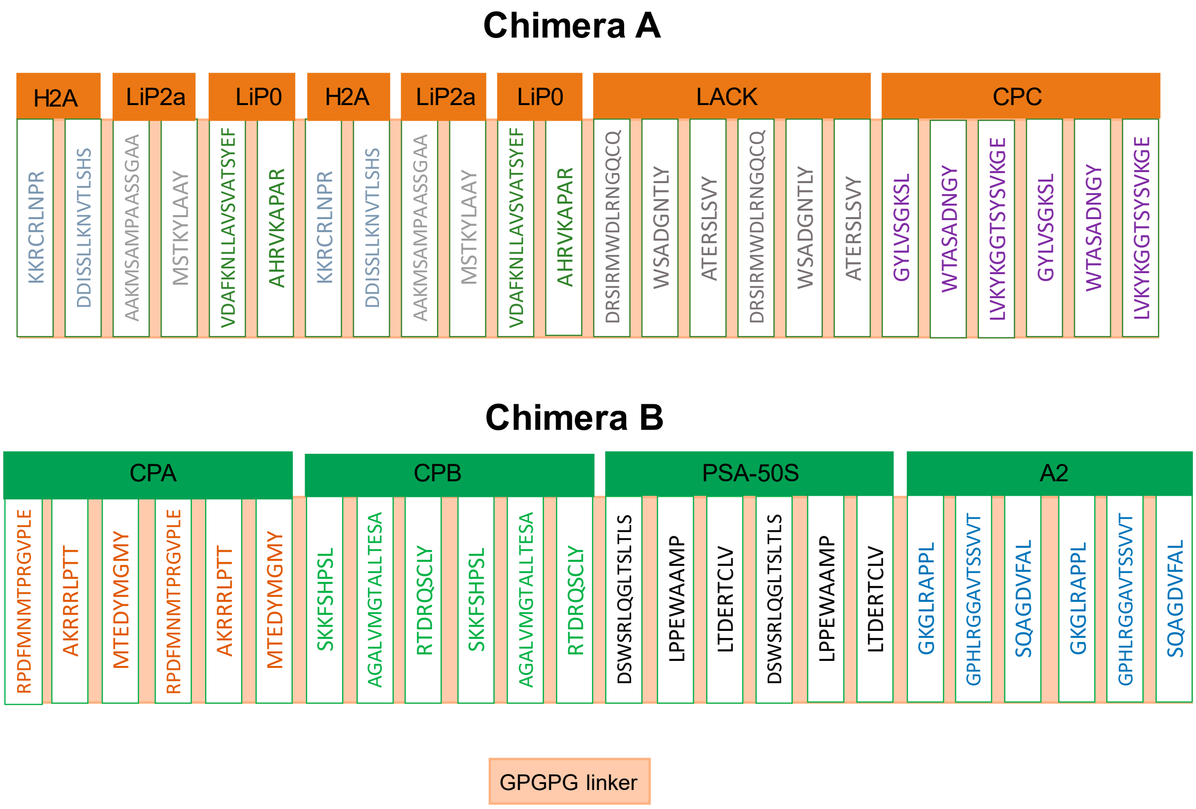

Figure S1. Illustration of the Chimera's constructions. The multi-epitope vaccine sequence consisting of 24 MHC class I and II ligands. GPGPG sequence was used as linker to join the epitopes. H2A= Histone protein 2, LiP2a = Acid ribosomal protein P2, LiP0 = Acid ribosomal protein P0, LACK = *Leishmania* homologue of activated C kinase, CPC = Cysteine peptidase C, CPA = Cysteine peptidase A, CPB = Cysteine peptidase B, PSA-50S = Surface antigenic protein, A2 = Amastigote protein A2

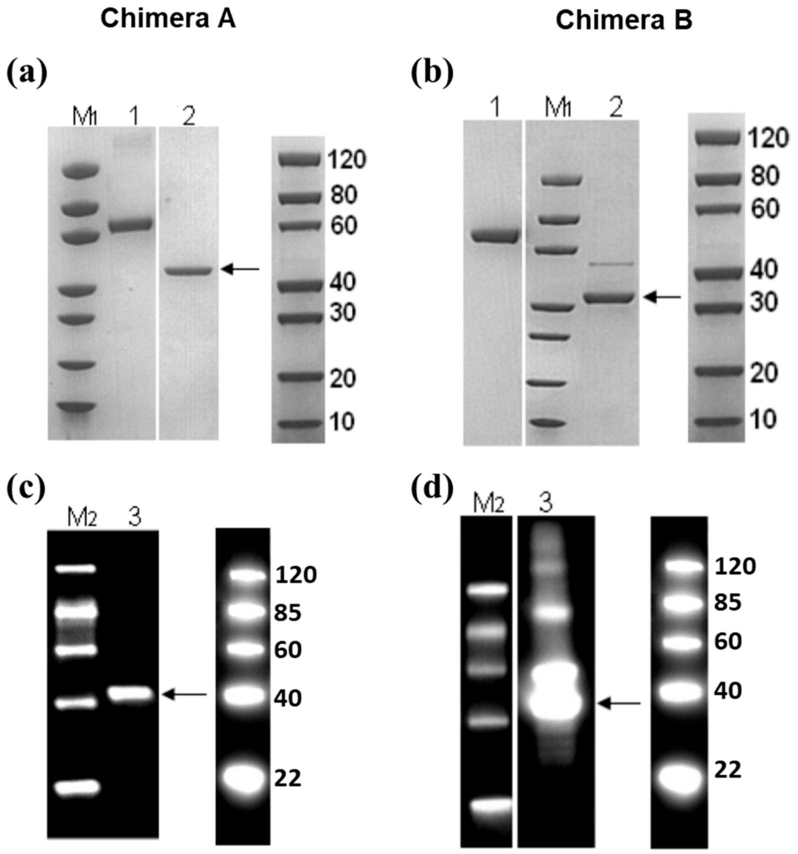

Figure S2. Evaluation of the expression of chimeric proteins A and B in the E. coli system. The expression was confirmed by SDS-PAGE as described in (a) and (b) for Chimeras A and B respectively. M1 = protein marker 1 (120 to 10 kDa); 1 = Bovine Serum Albumin (2 µg); 2 = 2 µg of chimeric protein A (~40 kDa) or B (~38 kDa). Also, the expression of the Chimera A (c) and Chimera B (d) was confirmed by Western Blot using anti-His antibody. M2 = protein marker 2 (120 to 22 kDa); 3 = 2 µg of chimeric protein A (c) and B (d).

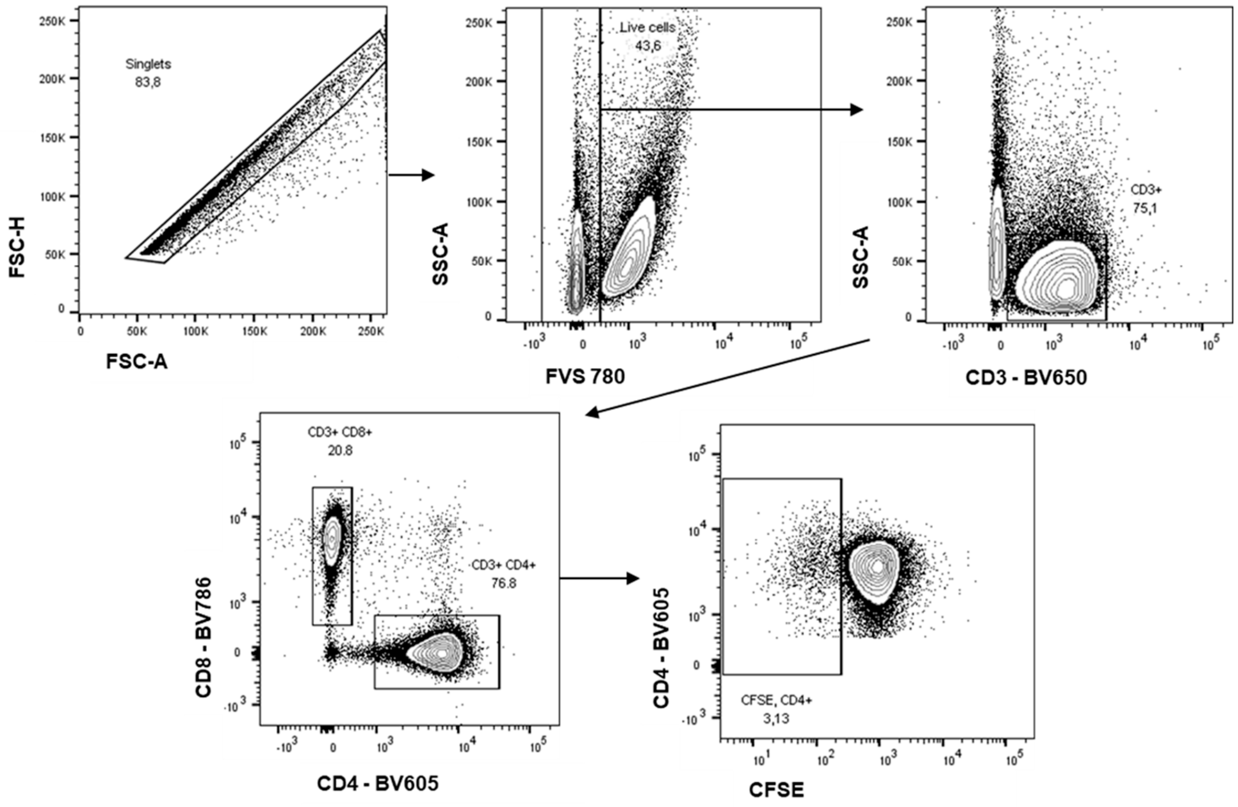

Figure S3. Representative plot of the gating strategy for CFSE-labelled cells to evaluate the proliferation of splenocytes. The first step was to select the single cells, then the live cells using (FVS780). To characterize the T-lymphocytes anti-CD3, anti-CD4 and anti-CD8 were used followed by the proliferation analyses selecting the CFSE^{low}-labelled cells.
